# Phonological productive processes in full-term schoolchildren and small for gestational age: a case-control study

**DOI:** 10.1590/2317-1782/20212020340

**Published:** 2021-12-20

**Authors:** Noemi Vieira de Freitas Rios, Luciene da Cruz Fernandes, Caio Leônidas Oliveira de Andrade, Ana Cecília Santiago, Crésio de Aragão Dantas Alves

**Affiliations:** 1 Curso de Fonoaudiologia, Departamento Ciências da Vida, Universidade Estadual da Bahia – UNEB - Salvador (BA), Brasil.; 2 Programa de Pós-graduação Processos Interativos dos Órgãos e Sistemas, Universidade Federal da Bahia – UFBA - Salvador (BA), Brasil.; 3 Departamento de Fonoaudiologia, Universidade Federal da Bahia – UFBA - Salvador (BA), Brasil.; 4 Departamento de Pediatria, Universidade Federal da Bahia – UFBA - Salvador (BA), Brasil.

**Keywords:** Speech Sound Disorder, Speech, Language and Hearing Sciences, Child Language Infant, Small for Gestational Age, Low Birth Weight

## Abstract

**Purpose:**

To characterize the use of phonological productive processes in a group of full-term children and small for gestational age and compare it with children appropriate for gestational age.

**Methods:**

Observational, analytical, case-control and non-paired study, nested in a cohort with the outcome of phonological disorder. We assessed 36 children according to the predetermined sample calculation, 24 (66.7%) without phonological disorders and 12 (33.3%) with phonological disorders. Of these, 24 (66.7%) children were classified as small for gestational age (SGA) and 12 (33%) as appropriate for gestational age (AGA). Phonological aspects of oral language were assessed by the ABFW children’s language test (2004). The results were subjected to descriptive analysis and, in order to assess the existence of an association among categorical variables, we used Fisher’s exact test for association.

**Results:**

The SGA group revealed a significantly higher number of phonological processes that change the syllable structure when compared to the AGA group. We noted that the phonological processes present and unexpected for age in the SGA population were: fricative plosivation, liquid simplification, palatal posteriorization and frontalization, plosive and fricative deafening, in addition to simplifying the consonant cluster and simplifying the final consonant, which were the most frequent in both groups.

**Conclusion:**

Although no association was found between phonological disorders and SGA children, we have noted a greater use of productive phonological processes in this group.

## INTRODUCTION

The development of oral verbal language is marked by simplifications of phonological rules in early childhood, where phoneme substitutions and omissions are the most common processes. This can generate strangeness in individuals with an already consolidated linguistic system, thus indicating the presence of a possible speech alteration, tolerable in younger children, but unacceptable at school age^(1)^.

When the typical developmental phonological processes (PP) do not disappear at the expected age and persist, or when atypical PP are present during the phonoaudiological assessment, the child is diagnosed with Speech Sound Disorder. This is considered the most prevalent communication disorder in the child population^(1,2)^. In Brazil, the prevalence can reach 9.17% in certain regions^(3)^.

When these disorders are not diagnosed early, they can directly interfere with school performance, mainly in the literacy process, as well as in the behavioral and psychosocial development of the child. Such damage may be perpetuated beyond the period of childhood^(4)^.

The auditory, visual, tactile, synesthetic and proprioceptive functions, the absence of alterations in the orofacial structures, in addition to the phonological memory, are essential for the adequate production of phonemes^(1,5)^. Studies reveal that, in several languages, phonemes follow an acquisition order and, initially, plosive and nasal phonemes emerge, followed by fricatives and liquids, in simple onset positions. This acquisition is completed around the age of five, reaching the most complex positions in the syllable, and may reach seven years. The variability of each language and individuality of the child should always be considered, with regard to the capacity of perception, cognition, production and interaction^(6-8)^.

Metabolic, cognitive, emotional and sensory alterations, as well as environmental differences, can interfere with speech development. Nevertheless, in many cases, specific organic or environmental causes are not identified. Among the aspects that have aroused the interest of researchers, one can mention prematurity and low weight, which are considered risk factors for language and speech alterations^(9-11)^.

Children considered small for gestational age (SGA) are the target of investigations because they present unfavorable conditions that appear to affect development^(12-14)^, in addition to constituting an important risk group due to the chance of morbidity and mortality^(14)^. These children have a birth weight lower than expected for their gestational age in weeks. This is most often considered for weight below the 10^th^ percentile, based on the intrauterine growth curve. This classification may be associated with intrauterine growth restriction (IUGR) or the baby may be constitutionally small due to genetic and/or physiological issues that determine this condition^(14)^. When birth weight is less than 2500g, in addition to SGA, it is classified as underweight^(15)^.

A systematic review and meta-analysis^(14)^ highlighted that full-term SGA children with IUGR presented significantly lower cognitive scores when compared to those appropriate for gestational age (AGA). Accordingly, the existence of possible impairments in the development of language and speech and the need for long-term follow-up should be considered. It is also observed a scarcity of studies in the researched literature characterizing these aspects in this population.

In light of the foregoing, this research sought to characterize the use of phonological processes in the group of children born full-term and small for gestational age, as well as to compare with those appropriate for gestational age, followed-up in an outpatient clinic for newborns in Salvador, Bahia, Brazil.

## METHODS

This is an observational, analytical, case-control and unpaired study, nested in a cohort with the phonological disorder outcome. “Case group” was defined as children with atypical phonological development (presence of phonological processes not expected for their age in any of the trials of the test used), while “control group” was defined as children with typical and appropriate phonological development for their age, coming from a population similar to the children grouped into “case”.

The sample composition of the cohort^(16)^ was considered and, as there was no such relationship, the sample size calculation was used and a high association of 2.7 was expected. Sample size calculation was used for an unpaired case-control study, defining: alpha: 5%; power: 80%; percentage of exposed (SGA) among “control” children: 12%; lowest expected value of the *odds-ratio*: 2.7; number of “control” children per case: 2, so the total number of “control” children is twice as high as the total number of “case” children.

After the phonoaudiological assessment and constitution of the case and control groups, the participants were classified, according to the adequacy of the birth weight for the referred gestational age, as: SGA, when the birth weight was less than the 10^th^ percentile; appropriate for gestational age (AGA), when born between the 10^th^ and 90^th^ percentiles, according to the INTERGROWTH-21st reference scale. Gestational age was defined according to the weeks of gestational age (information obtained from the date of the last menstruation, first trimester ultrasound or, in the absence of these, identified by the Capurro somatic or New Ballard method).

This study was approved by the Ethics Committee of the Climério de Oliveira Maternity Hospital, which belongs to the Federal University of Bahia (UFBA, as per its Portuguese acronym), under opinion nº 2.174.110/2017 and financed by the Research Support Foundation of the State of Bahia (FAPESB, as per its Portuguese acronym). All those responsible for the children signed the Free and Informed Consent Form (FICF).

### Participants and procedures

In order to make up the sample, children born full-term, served and followed-up in the outpatient clinics for SGA newborns (NB), high-risk NB and breastfed in public hospitals in the city of Salvador-BA, born in the same maternity hospitals during the same period, were included. These children were recruited from a cohort that followed-up children born SGA by means of a multidisciplinary team. This aimed to assess the issues of weight and height growth, body composition, hormonal, laboratory and neurodevelopmental assessment, as well as to investigate the relationships of these possible alterations in school age and the initial status.

The exclusion criteria adopted were: children with genetic syndrome, malformations, metabolic diseases, congenital infections, hearing alterations, concentration deficits and difficulties in understanding verbal commands. Those whose parents and/or guardians did not want to participate or who had no possibility of telephone contact for scheduling and those who did not attend the assessment were excluded.

Contact was made with family members/guardians of 55 full-term SGA and AGA children of school age between four and seven years, males and females, who were part of the study held in 2015^(16)^, and they were recalled to undergo pediatric, ophthalmological, nutritional, psychological and phonoaudiological assessment. Of these children, nine were excluded because they did not have active telephone contact and the others did not attend at least one of the assessments.

The phonoaudiological assessment consisted of an interview, auditory assessment, assessment of aspects related to speech and language (phonology, vocabulary and pragmatics) and assessment of the oral motor system and of the articulatory and bucofacial praxis.

Before applying the tests, the guardians of the children underwent an individual interview, where data on gestational history, pre, peri and/or postnatal events, neuropsychomotor and language development, socioemotional aspects, education and the child’s routine were collected. The participating children were assessed individually, in an appropriate environment, with no competitive stimuli that could undermine the quality of the assessment.

The assessment of the phonological aspects of oral language was performed using the ABFW (2004), a protocol indicated for children aged between 2 and 12 years, entirely directed to the Portuguese spoken in Brazil^(17)^. In order to exclude the existence of any organic factors that could hinder the production of speech sounds, the oral sensorimotor system was assessed using the Orofacial Myofunctional Evaluation with Scores (OMES Protocol), adapted for the objectives of the study^(18)^, in addition to the assessment of articulatory and bucofacial praxis. This corresponds to the execution of six movements of the lip, six of the tongue, six of the face and six articulatory movements, with 1 point being awarded for each movement correctly executed and no points (0) for those that were not executed^(19)^.

In order to assess the phonological linguistic level, 34 phonetically balanced figures were used for the naming test, 39 words for the imitation test and the analysis of a small excerpt of spontaneous speech, with a view to obtaining information about perceptual, cognitive-linguistic and motor production aspects of speech. The child’s production was recorded and filmed with a Sony camcorder and digital recorder, ICD PX333 model, and all assessments were carried out by a phonoaudiologist (speech therapist) with experience in child language disorders.

The assessments were blinded, since the examiner was previously unaware of the sample’s exposure factor. Subsequently, the data were phonetically transcribed into the specific test protocols and analyzed by two speech therapist judges. In all cases, there was a minimum agreement of 90% among the speech therapists.

The criteria and parameters suggested by the test were used, and then the traditional analysis of Portuguese language phonemes and phonological processes (PP) was performed. With the traditional analysis, the most frequent type of occurrence was checked, considering omissions, substitutions, distortions and correct answers, with 75% of correct answers being classified as acquired phoneme. The analysis by PP considered the productivity of each process, being classified as productive those that presented above 25% of occurrence in each of the tests and non-productive those that presented less than 25% of occurrence.

After analyzing the unexpected PP for the age and the constitution of the case and control groups, the analysis of the syllabic structure was also considered, according to the type of occurrence (substitution or omission), grouping PP in substitution processes (SP) and syllabic structure processes (SSP), often evidenced in complex syllable patterns^(20)^.

When considering the syllabic structure, the lateral and non-lateral liquid phonemes /ɾ/, /r/ and /l/, in addition to the fricative /s/, assume complex positions within the syllable. The consonant cluster simplification (CCS) PP can be considered a sociocultural marker, and it is very common to find the substitution of the consonant cluster of the phoneme /l/ by the phoneme /ɾ/. This alteration may not interfere with the understanding of the spoken message, as it is culturally accepted and varies according to region. Accordingly, it was decided to carry out a second analysis of the presence or absence of phonological alteration and the grouping of PP into SP and SSP, excluding the CCS process, as suggested by other authors^(21)^, discarding a variable of possible confusion, as it is one of the last PP to disappear in normal development and may represent a linguistic variation, not necessarily an alteration.

The socioeconomic classification was performed using the Brazilian economic classification criteria (CCEB, as per its Portuguese acronym) of the Brazilian Association of Companies and Research (ABEP, as per its Portuguese acronym)^(22)^. The classes are defined by CCEB, based on the instrument’s score, in A (45 to 100 points), B1 (38 to 44 points), B2 (29 to 37 points), C1 (23 to 28 points), C2 (17 to 22 points) and D-E (0 to 16 points). The authors simplified the classifications, grouping them into four classes based on the sample size. Therefore, classes A, B (score equivalent to classes B1 and B2), C (score equivalent to classes C1 and C2) and D-E were considered.

### Data analysis

The data from each group were tabulated according to the following categories: SGA or AGA exposure factor; gestational age in days; age of the child at the time of assessment in years; birth weight in grams; gender; CCEB classification; PP productivity in the imitation and naming tests; phoneme analysis in the naming and imitation tests. Subsequently, the data were analyzed by a qualified professional experienced in performing statistical analysis.

As for statistical analysis, it was performed using the Statistical Package for the Social Sciences (SPSS, version 21.0) software. The results were submitted to descriptive analysis using measures of central tendency and dispersion for continuous and discrete variables, as well as absolute and relative frequencies and percentage values for nominal and ordinal variables. In order to assess the existence of association among the categorical variables, the Fisher’s exact test of association was used. A significance level of 5% (p ≤ 0.05) was adopted, aiming to reject the null hypothesis with confidence intervals constructed with 95% statistical confidence.

## RESULTS

Of the 55 children who were part of the initial sample of the cohort, 36 of them were assessed according to the established sample calculation, with 24 (66.7%) with no phonological alterations and 12 (33.3%) with phonological alterations. Taking into account the exposure factor, 24 (66.7%) were classified as SGA and 12 (33%) as AGA (Table 1).

**Table 1:** Distribution of clinical and sociodemographic aspects in different study groups

***Clinical and social aspects***	Study groups					
	SGA			AGA		
	$\bar{X}$ (±SD)	n	%	$\bar{X}$ (±SD)	n	%
**Birth weight (g)**	2357.5(248.2)			3265.7(353.9)		
**Gestational age (days)**	274.3(8.4)			278,1(8.4)		
**Age (years)**	5.6 (0.9)			6,4 (0.7)		
**Age group (years)**						
04		02	8.3		00	0.0
05		10	41.7		01	8.3
06		07	29.2		05	41.7
07		05	20.8		06	50.0
***Gender***						
Male		13	54.2		06	50.0
Female		11	45.8		06	50.0
***Social class***						
Class A		00	0.0		00	0.0
Class B		04	16.7		00	0.0
Class C		18	75.0		11	91.7
Class D/E		02	8.3		01	8.3
***Language complaint***						
Absent		19	79.2		09	75.0
Present		05	20.8		03	25.0
***Phonological alteration***						
Absent		16	66.7		08	66.7
Present		08	33.3		04	33.3

Caption: SGA = small for gestational age; AGA = appropriate for gestational age; $\bar{X}$ (±SD) Average/Standard deviation; n = absolute frequency. Source: Designed by the authors themselves

The children’s age varied from four to seven years. There was a predominance of the age of five years, 10 (42%) in the SGA group ( $\bar{X}$ =5.6, SD±0.9), and the age of seven years, 6 (50%) in the AGA group ( $\bar{X}$ =6.4, SD±0.7). The groups were statistically different with regard to age (p= 0.013). As for gender,13 (54%) of the SGA children were males; in the AGA group, six (50%) were males. There was no statistical difference in gender distribution (p= 0.816).

With respect to birth weight, SGA children varied from 1,685 to 2,740g ( $\bar{X}$ =2,357, SD±248.2). In the AGA group, they varied from 2,684 to 3,825g ( $\bar{X}$ =3,265, SD±175.4) (Table 1).

Participants were classified into social classes, according to Table 1. It appears that most of the two groups belong to class C (SGA-75% and AGA-91.7%), and there was no statistical difference between the groups (p=0.291). As for the presence of complaints regarding the development of speech and language reported by the mothers during the interview, five (21%) children in the SGA group presented some type of complaint, compared to three (25%) in the AGA group, and this difference was not statistically significant between the groups (p= 0.078) (Table 1).

Through the spontaneous speech sample, it was identified that the assessed children presented an adequate communicative functional profile, actively participating in the proposed activities. No discrepancies were identified between the speech patterns in the test trials and the analyzed sample, as well as alterations in bucofacial and articulatory praxis.

The data distributed in Tables 2, 3 and 4 refer to the percentage of the number of children who presented each PP. When comparing the SGA and AGA groups, an association was observed between among SSP (p= 0.025). When comparing the genders, the presence of substitution PP (p= 0.045) and consonant cluster simplification PP (p = 0.022) was found to be significantly more predominant in males. (Table 2).

**Table 2:** Occurrence of phonological processes and the association among the study variables in the naming test

Phonological Processes	Gender (%)			Groups (%)			Language complaint (%)		
	Mal.	Fem.	p-value^a^	AGA	SGA	p-value^a^	Yes	No	p-value^a^
Fricative plosivation	0.0	5.9	0.290	0,0	4.2	0.480	12.5	0.0	0.061
Palatal posteriorization	10.5	11.7	0.907	0,0	16.7	0.139	37.5	3.6	0.008^*^
Palatal frontalization	10.5	0.0	0.175	0,0	8.3	0.310	25.0	0.0	0.007*
Liquid simplification	26.3	5.8	0.105	8,3	20.8	0.350	50.0	7.1	0.005*
Consonant cluster simplification	47.3	11.7	0.022*	25,0	33.3	0.614	75.0	17.9	0.002*
Final consonant simplification	31.6	17.6	0.342	8,3	33.3	0.107	75.0	10.7	0.000*
Devoicing of plosive	5.3	5.9	0.936	0,0	8.3	0.310	12.5	3.6	0.338
Devoicing of fricative	10.5	5.9	0.620	0,0	12.5	0.207	25.0	3.6	0.057
Affrication	5.3	17.6	0.487	8,3	8.3	1.000	25.0	3.6	0.057
Substitution processes	42.1	11.8	0.045*	8,3	33.3	0.107	87.5	14.3	0.000*
Syllabic Structure Processes	23.5	17.6	0.063	0,0	33.3	0.025*	62.5	25.0	0.050

a Fisher’s Exact test;

* p<0.005

Caption: SGA = small for gestational age; AGA = appropriate for gestational age; Mal. = male; Fem. = female. Source: Designed by the authors themselves

**Table 3:** Occurrence of phonological processes and the association among the study variables in the imitation test

Phonological Processes	Gender (%)			Groups (%)			Language complaint (%)		
	Mal.	Fem.	p-value^a^	AGA	SGA	p-value^a^	Yes	No	p-value^a^
Palatal posteriorization	10.5	5.9	0.620	0.0	12.5	0.207	37.5	0.0	0.001^*^
Palatal frontalization	10.5	0.0	0.175	0.0	8.3	0.310	25.0	0.0	0.007*
Liquid simplification	31.6	5.9	0.055	8.3	25.0	0.240	62.5	7.1	0.001*
Consonant cluster simplification	57.9	35.3	0.181	58.3	41.7	0.352	100.0	32.1	0.001*
Final consonant simplification	26.3	11.8	0.278	8.3	25.0	0.240	75.0	3.6	0.000*
Devoicing of plosive	5.3	5.9	0.936	0.0	8.3	0.310	12.5	3.6	0.338
Devoicing of fricative	10.5	11.8	0.907	8.3	12.5	0.712	25.0	7.1	0.162
Affrication	10.5	0.0	0.175	0.0	8.3	0.310	25.0	0.0	0.007*
Substitution processes	47.4	29.4	0.277	16.7	29.2	0.421	100.0	21.4	0.000*
Syllabic Structure Processes	36.8	47.4	0.393	0.0	20.8	0.093	62.5	17.9	0.014*

a Fisher’s Exact test;

* p<0.005

Caption: SGA = small for gestational age; AGA = appropriate for gestational age; Mal. = male; Fem. = female. Source: designed by the authors themselves

**Table 4:** Analysis of omissions and substitutions in consonant clusters and final consonants between groups and in the naming and imitation tests

Types of occurrence		Naming			Imitation		
		SGA (%)	AGA (%)	p-value^a^	SGA (%)	AGA (%)	p-value^a^
Omission	FC	18.8	8.3	0.654	22.9	8.3	0.607
	CC	25.6	2.4		19.4	1.4	
Substitution	FC	2	4.1	0.327	10.4	8.3	0.608
	CC	11.3	11.4		9	18.8	

a Fisher’s Exact test;

Caption: SGA = small for gestational age; AGA = appropriate for gestational age; FC = final consonant; CC = consonant cluster. Source: Designed by the authors themselves

The results related to the most of PP and the initial language complaint presented by the parents in the interview, during the naming and imitation tasks, were considered significant. Regarding the other variables, it was noted an absence of association (Tables 2 and 3).

As for the analysis of the frequency of occurrences of phonological processes in the imitation and naming tests between the groups, no statistical difference was found, but it was observed that the average of occurrence for all processes present in the population always presented a higher percentage in the SGA group.

**Figure 1:**
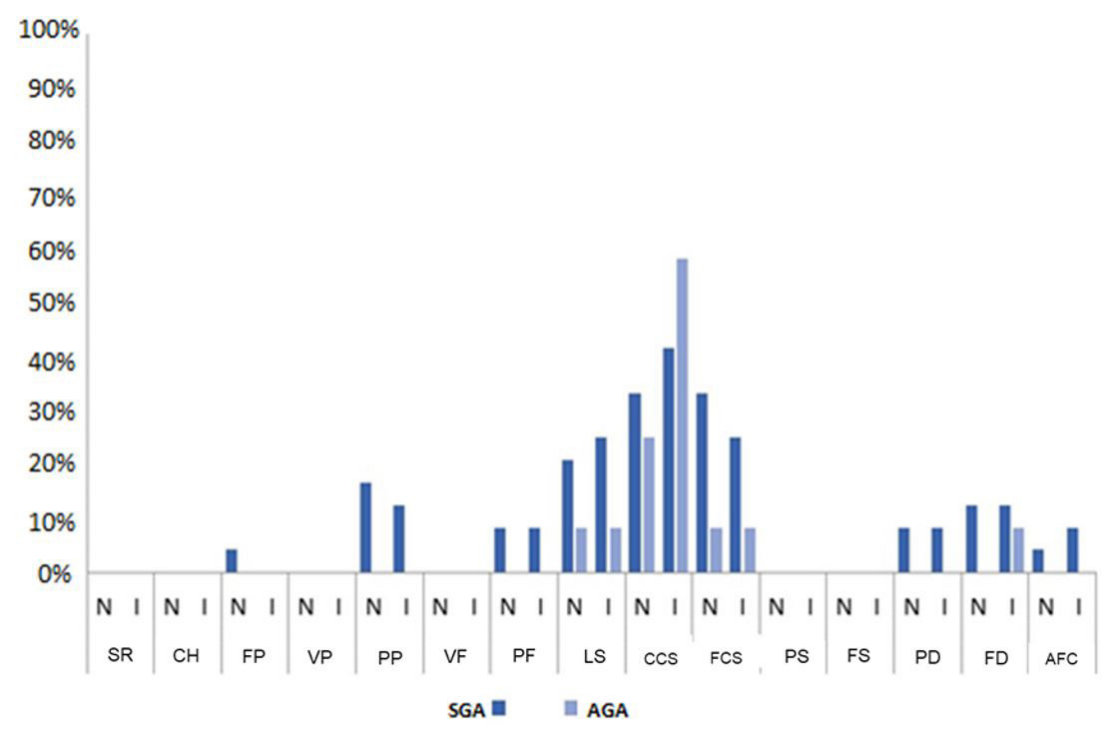
Comparison and occurrence of productive phonological processes in the naming and imitation tests between the SGA and AGA groups

As for the presence of productive PP, of the 14 processes analyzed in the naming test, the SGA group presented alterations in eight processes. In the current research, the affrication (palatalization) PP was added, not observed in typical child development, totaling nine altered processes. The AGA group presented alterations in only three processes. In both tests, the highest prevalence in the SGA group stands out, except in the CCS process in the imitation test (Figure 1).

Caption: SGA = small for gestational age; AGA = appropriate for gestational age; N = naming test; I = imitation test; SR = syllable reduction; CH = consonant harmony; FP = fricative plosivation; VP = velar posteriorization; PP = palatal posteriorization; VF =velar frontalization; PF = palatal frontalization; LS = liquid simplification; CCS = consonant cluster simplification; FCS = final consonant simplification; PS = plosive sounds; FS = fricative sounds; PD = devoicing of plosive; FD = devoicing of fricative; AFC = affrication. Source: Designed by the authors themselves

Table 4 analyzes the most frequent type of occurrence considering the omissions and substitutions of lateral and non-lateral liquid phonemes in the more complex syllabic structures consisting of consonant clusters and final consonants. The predominance of omissions of lateral and non-lateral liquid phonemes was identified in the SGA group in consonant clusters and in final consonants.

## DISCUSSION

According to the results found, it appear that the studied population presents a significantly higher number of phonological processes that alter the syllable structure when compared to the SGA group, in addition to presenting a greater use of productive and unexpected PP for their age. It should be underlined that alterations in the phonological aspect can be considered one of the most evident in child development. In this context, early diagnosis and intervention become essential before intercurrences in the learning of reading and writing are installed, as well as psychosocial implications that may interfere directly with the perception of mother tongue speaker, entailing negative impacts.

These data suggest that differences regarding the presence of phonological processes that alter the syllabic structure between the SGA and AGA groups can be justified by the difference in age between the groups, as well as by pathophysiological issues in the studied population, which appear to limit the production of words with greater articulation demand, as the errors of the consonant cluster in the SGA group were predominantly due to omission, and not substitution, as in the AGA group (Table 4). A recent study^(14)^ highlighted that SGA children who had IUGR presented alterations in cognitive development when compared to AGA children, which could justify the possible alterations in phonological development, mainly regarding the organization of more complex syllabic structures within the word, which is a process that involves the interaction of linguistic, auditory and cognitive skills, and not exclusively the articulatory skill.

A possible placental dysfunction exposes the baby to malnutrition, decreased growth rate and, probably, affects the processes of brain development, thus triggering structural and functional alterations, situations commonly observed in babies who had IUGR associated or not with prematurity, which potentially highlights the emergence of altered neurological and cognitive processes^(23)^, which can interfere with phonological processing^(11)^. Another study^(13)^ reported limited evidence that children with IUGR who were born full-term SGA may have problems with attention, impacting learning and behavior as they progress through school, thus suggesting that this would be a useful area for future work.

In the current study, children were classified as SGA when relating weight and GA soon after birth. As this classification is widely discussed in the literature because it contains pathophysiological uncertainties, in this research, as in others related to the topic, there is a limitation in the classification and in the definition if the child is SGA because of having suffered an IUGR or if it is constitutionally small, because of genetic and/or physiological issues that determine this condition, and not because of pathological issues.

The phonological processes of CCS and FCS were the most present in the studied population, corroborating studies^(24-26)^ that also reported that, as they involve more complex syllabic structures, they are the last to disappear. The maximum expected age for the elimination of these processes, according to the test used, would be seven years^(17)^, with studies warning of earlier disappearance^(8,25,27)^. It should even be highlighted that the study conducted with children from Salvador^(28)^, Bahia, Brazil, and others refer to the possibility of the influence of linguistic variation on phonological acquisition, thus interfering with the age of elimination of these processes^(7,21,29)^.

When analyzing the investigated age group, the end of the acquisition period is expected to happen with the stabilization of errors and predominance of phonological accuracy starting at the age of five^(8,24,28)^. In the SGA group, the average age was lower than the AGA group, but above five years in both groups. Even in the face of this difference, the number of unexpected production processes for their age is more marked in the SGA group, as displayed in Figure 1.

The presence of PP was identified as fricative plosivation and liquid simplification, which, according to the test used, the age of disappearance is between two and three and a half years, in addition to palatal posteriorization and palatal frontalization, where elimination takes place by around four and a half years^(17)^. Moreover, it should be underlined that the presence of PP that are not common in typical phonological development, such as devoicing PP; and, in the specific case of this sample, the presence of affrication PP also took place.

A study conducted in 2012^(26)^ found that the phonological processes of CCS and FCS were present in the age group between 7 years and 7 years and 11 months, especially in children from low economic classes. The authors of this study question the presence of PP at older ages, since the ideal condition, especially in the first grade of *ensino fundamental* I (early stage of elementary school in Brazil), which corresponds to the age group of six and seven years, is that children do not present any type of phonological alreation in their speech, as the writing of words is initially based on orality^(30)^. This reinforces the need for early detection and intervention of speech disorders, regardless of the investigated population.

It should be underlined that the groups were not paired with regard to social class and gender; however, both groups presented similar exposure to socioeconomic and cultural aspects, considered important risk factors that influence phonological development^(24)^. Added to this, there was no association between these variables and the groups.

In the sample, similar distributions between the genders were observed; however, a greater proportion of phonological disorders was found in males, thus confirming other studies^(3,7,24)^. Regarding the CCS phonological process in the naming test, it was noted that the occurrence was significantly more present in boys than in girls (p = 0.022)^(31)^, disagreeing with the study that observed a tendency for boys to present greater phonological accuracy.

An association was detected between the complaint of possible alterations in the children’s speech and most of the phonological processes (Table 2), confirming that the parents perceive the presence of PP. These parents appear to identify speech production as inappropriate, although acceptable, or common, in their social environment because, in most cases, it does not interfere with the understanding of the message^(21,29)^, as happens with the PP of LS, CCS and FCS, who presented higher occurrence in the studied population. It is believed that this perception of parents is related to the search for a culturally accepted speech pattern and that, if the child achieves it, he/she has possibilities for changes that will favor the confrontation of other communicative spaces, without running the risk that society will label their children as those “who don’t know how to speak or who speak wrongly”. It is understood that achieving a more appropriate speech pattern alleviates psychosocial and emotional distress^(1)^, which is possibly the goal these parents seek for their children.

Regarding the finding that the AGA group presents a higher average of the CCS PP than the SGA group, as well as the types of errors, it was perceived that the predominance is the substitution of the lateral liquid /l/ for the non-lateral liquid /ɾ/, leading to think that it would indeed be a linguistic variation, and not a lack of skill in the production of more complex syllables, thus corroborating other authors^(21,27)^.

This theme is still little explored, and thus studies with more significant samples are needed, covering other aspects of language, in addition to the phonological context.

The results of this study serve as a warning to health professionals who work with this population in relation to the need for prevention, identification and early intervention of speech disorders, aiming at healthy communication and quality of life. It is also essential to pay more attention to communication health in early childhood, with the implementation of public policies that include children who are born full-term and are classified as SGA, in order to avoid future damage in areas that affect cognitive, linguistic, psychological and social development.

Although no association was observed between phonological alterations and SGA children, there were particularities in some phonological aspects of this population, which deserves to be further investigated, including studies with a larger number of children, since an important limitation was the small sample size of this cohort. This even made it impossible to match the children by age. It is believed that, with a larger sample, the evidence related to the higher number of productive processes in the SGA group will be statistically confirmed.

Another consideration is that the classification used does not make it possible to make differentiations among children who are SGA because of some kind of suffering during the intrauterine period and those who are small due to genetic and/or physiological reasons, excluding any pathological condition.

## CONCLUSION

In the present study, it was observed that SGA children presented a significantly greater number of phonological processes that alter the syllable structure when compared to those in the AGA group. The present and unexpected phonological processes for age in the SGA population were fricative plosivation, liquid simplification, palatal posteriorization and palatal frontalization, devoicing of plosive and fricative, in addition to consonant cluster simplification and final consonant simplification, which were the most frequent occurrence in both groups. No association was found between phonological alterations and SGA children; however, a greater use of productive phonological processes was identified in this group. It should be highlighted the importance of a differentiated look at this population, in order to detect early alterations related to aspects of oral language such as phonology.
