# Supplementary material for: Phonological productive processes in full-term schoolchildren and small for gestational age: a case-control study
Source: Codas. 2021 Dec 20;34(2):e20200340. doi: 10.1590/2317-1782/20212020340 (PMC9851191; doi:10.1590/2317-1782/20212020340)
Supplement: Translation

# Translation: Processos fonológicos produtivos em escolares nascidos a termo e pequenos para a idade gestacional: estudo caso-controle

**Authors:** Noemi Vieira de Freitas Rios, Luciene da Cruz Fernandes, Caio Leônidas Oliveira de Andrade, Ana Cecília Santiago, Crésio de Aragão Dantas Alves

**Affiliations:** 1 Curso de Fonoaudiologia, Departamento Ciências da Vida, Universidade Estadual da Bahia – UNEB - Salvador (BA), Brasil.; 2 Programa de Pós-graduação Processos Interativos dos Órgãos e Sistemas, Universidade Federal da Bahia – UFBA - Salvador (BA), Brasil.; 3 Departamento de Fonoaudiologia, Universidade Federal da Bahia – UFBA - Salvador (BA), Brasil.; 4 Departamento de Pediatria, Universidade Federal da Bahia – UFBA - Salvador (BA), Brasil.

**Keywords:** Transtorno Fonológico, Fonoaudiologia, Linguagem Infantil, Recém-Nascido Pequeno para a Idade Gestacional, Baixo Peso ao Nascer

## Abstract

**Objetivo:**

Caracterizar o uso de processos fonológicos produtivos no grupo de crianças nascidas a termo e pequenas para a idade gestacional e compará-lo com crianças adequadas para a idade gestacional.

**Método:**

Estudo observacional, analítico, do tipo caso-controle, não pareado, aninhado a uma coorte com o desfecho alteração fonológica. Foram avaliadas 36 crianças de acordo com o cálculo amostral pré-estabelecido, sendo 24(66,7%) sem alterações fonológicas e 12(33,3) com alteração fonológica. Dessas, 24(66,7%) crianças foram classificadas como pequeno para a idade gestacional (PIG) e 12(33%), como adequada para a idade gestacional (AIG). Os aspectos fonológicos da linguagem oral foram avaliados pelo teste de linguagem infantil ABFW (2004). Os resultados foram submetidos à análise descritiva e a fim de avaliar a existência de associação entre as variáveis categóricas, foi utilizado o teste exato de Fisher de associação.

**Resultados:**

O grupo PIG apresentou significativamente maior número de processos fonológicos que alteram a estrutura da sílaba quando comparado ao grupo AIG. Observou-se que os processos fonológicos presentes e não esperados para idade na população PIG foram: plosivação de fricativa, simplificação de líquidas, posteriorização e frontalização de palatal, ensurdecimento de plosivas e fricativas, além da simplificação do encontro consonantal e simplificação de consoante final, que foram os de maior ocorrência em ambos os grupos.

**Conclusão:**

Embora não tenha sido encontrada associação entre alterações fonológicas e crianças PIG, observou-se maior uso de processos fonológicos produtivos neste grupo.

## INTRODUÇÃO

O desenvolvimento da linguagem verbal oral é marcado por simplificações das regras fonológicas na primeira infância, sendo as substituições e omissões de fonemas os processos mais comuns. Isso pode gerar estranhamento nos indivíduos com o sistema linguístico já consolidado, indicando a presença de uma possível alteração de fala, tolerável em crianças menores, porém inaceitável na idade escolar^(1)^.

Quando os processos fonológicos (PF) típicos do desenvolvimento não desaparecem na idade esperada e persistem ou quando PF atípicos estão presentes durante a avaliação fonoaudiológica, a criança é diagnosticada com Transtorno dos Sons da Fala. Este é considerado o distúrbio da comunicação mais prevalente na população infantil^(1,2)^. No Brasil, a prevalência pode chegar a 9,17% em determinadas regiões^(3)^.

Quando esses transtornos não são diagnosticados precocemente, podem interferir diretamente no rendimento escolar, principalmente no processo de alfabetização, comportamento e no desenvolvimento psicossocial da criança. Tais danos podem perpetuar para além da infância^(4)^.

A função auditiva, visual, tátil sinestésica, proprioceptiva e a ausência de alterações nas estruturas orofaciais, além da memória fonológica, são essenciais para a produção adequada dos fonemas^(1,5)^. Estudos revelam que em várias línguas os fonemas seguem uma ordem de aquisição e inicialmente aparecem os fonemas plosivos e nasais, seguidos dos fricativos e líquidos, nas posições de *onset* simples. Essa aquisição é finalizada por volta dos cinco anos, alcançando as posições mais complexas na sílaba, podendo chegar aos sete anos. Considera-se sempre a variabilidade de cada língua e individualidade da criança, no que se refere à capacidade de percepção, cognição, produção e interação^(6-8)^.

Alterações metabólicas, cognitivas, emocionais e sensoriais, bem como diferenças ambientais, podem interferir no desenvolvimento da fala. Todavia, em muitos casos, não se identificam causas específicas orgânicas ou ambientais. Entre os aspectos que têm despertado interesse nos pesquisadores estão a prematuridade e o baixo peso, fatores considerados de risco para alterações de linguagem e fala^(9-11)^.

As crianças consideradas pequenas para a idade gestacional (PIG) são alvo de investigações por apresentar condições desfavoráveis que parecem afetar o desenvolvimento^(12-14)^, além de constituir um importante grupo de risco devido à chance de morbimortalidade^(14)^. Essas crianças apresentam peso ao nascimento abaixo do esperado para respectiva idade gestacional em semanas. Isso é considerado, com mais frequência, para o peso situado abaixo do percentil 10, com base na curva de crescimento intrauterino. Essa classificação pode estar associada à restrição do crescimento intrauterino (RCIU) ou o bebê pode ser constitucionalmente pequeno por questões genéticas e/ou fisiológicas que determinam essa condição^(14)^. Quando o peso ao nascer é inferior a 2500g, além de PIG, é classificado como baixo peso^(15)^.

Revisão sistemática e meta-análise^(14)^ destacou que crianças PIG nascidas a termo com RCIU apresentaram escores cognitivos significativamente menores quando comparado àquelas adequadas para a idade gestacional (AIG). Assim sendo, considera-se a existência de possíveis comprometimentos no desenvolvimento da linguagem e fala e a necessidade do acompanhamento em longo prazo. Observa-se também uma escassez de estudos na literatura pesquisada que caracterizam esses aspectos nessa população.

Diante do exposto, a presente pesquisa buscou caracterizar o uso de processos fonológicos no grupo de crianças nascidas a termo e pequenas para a idade gestacional, como também comparar com aquelas adequadas para a idade gestacional, acompanhadas em um ambulatório de assistência ao recém-nascidos em Salvador, Bahia, Brasil.

## MÉTODO

Trata-se de um estudo observacional, analítico, do tipo caso-controle, não pareado, aninhado a uma coorte com o desfecho alteração fonológica. Definiu-se caso as crianças que apresentaram desenvolvimento fonológico atípico (presença de processos fonológicos não esperados para a idade em qualquer uma das provas do teste utilizado) e controle as crianças que apresentaram desenvolvimento fonológico típico e adequado para a idade, vindo de população semelhante à dos casos.

Considerou-se a composição amostral da coorte^(16)^ e, por não ter essa relação, o cálculo amostral foi utilizado e esperada uma associação alta de 2,7. Utilizou-se o cálculo do tamanho de amostra para estudo de caso-controle não pareado, definindo-se: alfa: 5%; poder: 80%; porcentagem de expostos (PIG) entre os controles:12%; menor valor esperado da *odds-ratio*: 2,7; número de controles por caso: 2, sendo então o total de controles duas vezes maior que o total de casos.

Após a avaliação fonoaudiológica e constituição dos grupos caso e controle, os participantes foram classificados, de acordo com a adequação do peso de nascimento para a referida idade gestacional, como: PIG, quando o peso de nascimento foi menor que o percentil 10; adequado para a idade gestacional (AIG), quando nascido entre o percentil 10 e 90, segundo a escala de referência INTERGROWTH-21^st^. A idade gestacional foi definida conforme as semanas de idade gestacional (informação obtida a partir da data da última menstruação, ultrassonografia do primeiro trimestre ou, na ausência destas, identificada pelo método de Capurro somático ou *New Ballard*).

Este estudo foi aprovado pelo Comitê de Ética da maternidade Climério de Oliveira da Universidade Federal da Bahia (UFBA) sob o nº 2.174.110/2017 e financiado pela Fundação de Amparo à Pesquisa da Bahia (FAPESB). Todos os responsáveis pelas crianças assinaram o Termo de Consentimento Livre e Esclarecido (TCLE).

### Participantes e procedimentos

Para a constituição da amostra, incluíram-se as crianças nascidas a termo, atendidas e acompanhadas nos ambulatórios de *follow-up* de recém-nascidos (RN) PIG, RN de alto risco e de aleitamento materno de hospitais públicos da cidade de Salvador-BA, nascidas nas mesmas maternidades clínicas, durante o mesmo período. Essas crianças foram recrutadas de uma coorte que realizou o acompanhamento de crianças nascidas PIG por uma equipe multidisciplinar. Esta visava avaliar as questões de crescimento pondero-estatural, composição corporal, avaliação hormonal, laboratorial e neurodesenvolvimento, bem como investigar as relações dessas possíveis alterações na idade escolar e o quadro inicial.

Adotaram-se como critérios de exclusão crianças com síndrome genética, malformações, doenças metabólicas, infecções congênitas, alterações auditivas, *deficit* de concentração e dificuldades para entender comandos verbais. Excluíram-se aquelas cujos pais e/ou responsáveis não quiseram participar ou que não houve possibilidade de contato telefônico para o agendamento e as que não compareceram à avaliação.

Fez-se contato com os familiares/responsáveis por 55 crianças nascidas a termo PIG e AIG, de ambos os gêneros, que fizeram parte do estudo de 2015^(16)^, convocadas novamente na idade escolar entre quatro a sete anos para a realização de avaliação pediátrica, oftalmológica, nutricional, psicológica e fonoaudiológica. Dessas crianças, nove foram excluídas por não apresentar contato telefônico ativo e as demais não compareceram a pelo menos uma das avaliações.

A avaliação fonoaudiológica foi constituída por entrevista, avaliação auditiva, avaliação de aspectos relacionados à fala e linguagem (fonologia, vocabulário e pragmática) e avaliação do sistema motor oral e das praxias articulatórias e bucofacias.

Antes da aplicação dos testes, os responsáveis pelas crianças foram submetidos a uma entrevista individual na qual foram coletados dados sobre antecedentes gestacionais, intercorrências pré, peri e/ou pós-natal, desenvolvimento neuropsicomotor e de linguagem, aspectos socioemocionais, escolaridade e rotina da criança. As crianças participantes foram avaliadas individualmente, em ambiente adequado, com ausência de estímulos competitivos que pudessem comprometer a qualidade da avaliação.

A avaliação dos aspectos fonológicos da linguagem oral foi realizada por meio do ABFW (2004), protocolo indicado para crianças de dois a 12 anos, inteiramente direcionado para o português falado no Brasil^(17)^. Com o intuito de excluir a existência de quaisquer fatores orgânicos que pudessem impedir a produção dos sons da fala, realizou-se a avaliação do sistema sensório-motor oral utilizando-se o Protocolo de Avaliação Miofuncional Orofacial com Escores (AMIOFE), adaptado para os objetivos do estudo^(18)^, somado à avaliação das praxias articulatórias e bucofaciais. Isso corresponde à realização de seis movimentos de lábio, seis de língua, seis de face e seis articulatórios, sendo atribuído 1 ponto para cada movimento executado corretamente e nenhum ponto (0) para aqueles que não foram executados^(19)^.

Para a avaliação do nível linguístico fonológico, utilizaram-se as 34 figuras foneticamente balanceadas para a prova de nomeação, 39 vocábulos para a prova de imitação e a análise de um pequeno trecho de fala espontânea com o intuito de obter informações sobre os aspectos perceptivo, cognitivo-linguístico e da produção motora da fala. A produção da criança foi gravada e filmada com filmadora e gravador digital da marca Sony, modelo ICD PX333, sendo todas as avaliações realizadas por fonoaudióloga com experiência em transtornos da linguagem infantil.

As avaliações foram cegas, uma vez que a examinadora desconhecia previamente o fator de exposição da amostra. Posteriormente, os dados foram transcritos foneticamente nos protocolos específicos do teste e analisados por dois juízes fonoaudiólogos. Em todos os casos, houve acordo mínimo de 90% entre os fonoaudiólogos.

Utilizaram-se os critérios e parâmetros sugeridos pelo teste e realizou-se a análise tradicional dos fonemas da língua portuguesa e dos processos fonológicos (PF). Com a análise tradicional, verificou-se o tipo de ocorrência mais frequente, considerando as omissões, substituições, distorções e acertos, sendo classificado como fonema adquirido aquele que apresentou 75% de acerto. A análise por PF considerou a produtividade de cada processo, sendo classificado como produtivos os que apresentaram acima de 25% de ocorrência em cada uma das provas e não-produtivos os que apresentaram menos de 25% de ocorrência.

Após a análise dos PF não esperados para a idade e constituição dos grupos caso e controle, considerou-se também a análise da estrutura silábica, de acordo com o tipo de ocorrência (substituição ou omissão), agrupando os PF em processos de substituição (PS) e processos de estruturação silábica (PES), frequentemente evidenciados nos padrões de sílabas complexas^(20)^.

Ao considerar a estrutura silábica, os fonemas líquidos laterais e não laterais /ɾ/, /r/ e /l/, além do fricativo /s/, assumem posições complexas dentro da sílaba. O PF de simplificação do encontro consonantal (SEC) pode ser considerado um marcador sociocultural, sendo muito comum encontrar a substituição do encontro consonantal do fonema/l/ pelo fonema /ɾ/. Essa alteração pode não interferir na compreensão da mensagem falada, é culturalmente aceita e varia de acordo com a região. Sendo assim, optou-se em realizar uma segunda análise da presença ou ausência de alteração fonológica e o agrupamento dos PF em PS e PES excluindo o processo SEC, como sugerido por outros autores^(21)^, descartando uma variável de possível confusão, já que é um dos últimos PF a desaparecer no desenvolvimento normal e pode representar uma variação linguística, não necessariamente uma alteração.

A classificação socioeconômica foi realizada utilizando-se os critérios de classificação econômica brasileira (CCEB) da Associação Brasileira de Empresas e Pesquisas (ABEP)^(22)^. As classes são definidas pelo CCEB, a partir da pontuação do instrumento, em A (45 a 100 pontos), B1 (38 a 44 pontos), B2 (29 a 37 pontos), C1 (23 a 28 pontos), C2 (17 a 22 pontos) e D-E (0 a 16 pontos). Os autores simplificaram as classificações, agrupando-as em quatro classes em decorrência do tamanho da amostra. Logo, consideram-se as classes A, B (pontuação equivalente às classes B1 e B2), C (pontuação equivalente às classes C1 e C2) e D-E.

### Análise de dados

Os dados de cada grupo foram tabulados de acordo com as seguintes categorias: fator exposição PIG ou AIG; idade gestacional em dias; idade da criança na data da avaliação em anos; peso ao nascer em gramas; sexo; classificação CCEB; produtividade dos PF nas provas de imitação e nomeação; análise dos fonemas nas provas de nomeação e imitação. Posteriormente, os dados foram analisados por um profissional qualificado com experiência na realização de análise estatística.

Para a análise estatística utilizou-se o *software computacional Statistical Package for Social Sciences* (SPSS, versão 21.0). Os resultados foram submetidos à análise descritiva por meio de medidas de tendência central e dispersão para as variáveis contínuas e discretas, bem como as frequências absolutas, relativas e valores percentuais para as variáveis nominais e ordinais. A fim de avaliar a existência de associação entre as variáveis categóricas, o teste exato de Fisher de associação foi utilizado. Adotou-se um nível de significância de 5% (p ≤ 0,05) visando rejeitar a hipótese de nulidade com intervalos de confiança construídos com 95% de confiança estatística.

## RESULTADOS

Das 55 crianças que fizeram parte da amostra inicial da coorte, avaliaram-se 36 delas de acordo com o cálculo amostral estabelecido, sendo 24(66,7%) sem alterações fonológicas e 12(33,3) com alteração fonológica. Levando em consideração o fator de exposição, 24 (66,7%) foram classificadas como PIG e 12 (33%) como AIG (Tabela 1).

**Tabela 1:** Distribuição dos aspectos clínicos e sociodemográficos nos diferentes grupos de estudo

***Aspectos Clínicos e sociais***	Grupos de estudo					
	PIG			AIG		
	$\bar{X}$ (±DP)	n	%	$\bar{X}$ (±DP)	n	%
**Peso ao nascer (g)**	2357,5(248,2)			3265,7(353,9)		
**Idade gestacional (dias)**	274,3(8,4)			278,1(8,4)		
**Idade (anos)**	5,6 (0,9)			6,4 (0,7)		
**Faixa etária(anos)**						
04		02	8,3		00	0,0
05		10	41,7		01	8,3
06		07	29,2		05	41,7
07		05	20,8		06	50,0
***Sexo***						
Masculino		13	54,2		06	50,0
Feminino		11	45,8		06	50,0
***Classe social***						
Classe A		00	0,0		00	0,0
Classe B		04	16,7		00	0,0
Classe C		18	75,0		11	91,7
Classe D/E		02	8,3		01	8,3
***Queixa de linguagem***						
Ausente		19	79,2		09	75,0
Presente		05	20,8		03	25,0
***Alteração fonológica***						
Ausente		16	66,7		08	66,7
Presente		08	33,3		04	33,3

Legenda: PIG = pequenos para idade gestacional; AIG = adequados para idade gestacional; $\bar{X}$ (±DP) média/Desvio padrão; n = frequência absoluta. Fonte: Própria

A idade das crianças variou de quatro a sete anos. Observou-se o predomínio da idade de cinco anos, 10 (42%) no grupo PIG ( $\bar{X}$ =5,6, DP±0,9), e da idade de sete anos, 6(50%) no grupo AIG ( $\bar{X}$ =6,4, DP±0,7). Os grupos foram estatisticamente diferentes no que se refere à idade (p= 0,013). Quanto ao sexo,13 (54%) das crianças PIG eram do sexo masculino; no grupo AIG, seis (50%) eram do sexo masculino. A distribuição do sexo não teve diferença estatística (p= 0,816).

No que tange ao peso ao nascer, as crianças PIG apresentaram variação de 1685 a 2740 g ( $\bar{X}$ =2357, DP±248,2). No grupo AIG, variação de 2684 a 3825g ( $\bar{X}$ =3265, DP±175,4) (Tabela 1).

Os participantes foram classificados em classes sociais, de acordo com a Tabela 1. Constata-se que a maioria dos dois grupos pertence à classe C (PIG-75% e AIG-91.7%), sendo que não teve diferença estatística entre os grupos (p=0,291). Quanto à presença de queixas com relação ao desenvolvimento da fala e linguagem relatadas pelas mães durante a entrevista, cinco (21%) crianças do grupo PIG apresentaram algum tipo de queixa, comparadas a três (25%) do grupo AIG, sendo que essa diferença não foi estatisticamente significante entre os grupos (p= 0,078) (Tabela 1).

Por meio da amostra de fala espontânea, identificou-se que as crianças avaliadas apresentaram perfil funcional comunicativo adequado, participando ativamente das atividades propostas. Não foram identificadas discrepâncias entre os padrões de fala nas provas do teste e a amostra analisada, como também alterações nas praxias bucofaciais e articulatórias.

Os dados distribuídos nas Tabelas 2, 3 e 4 referem-se à porcentagem do número de crianças que apresentavam cada PF. Na comparação entre os grupos PIG e AIG, observou-se associação entre o PES (p= 0,025). Quando comparados os sexos, encontrou-se a presença de PF de substituição (p = 0,045) e o PF de simplificação do encontro consonantal (p= 0,022) significativamente mais predominante no sexo masculino (Tabela 2).

**Tabela 2:** Ocorrência dos processos fonológicos e a associação entre as variáveis do estudo na prova de nomeação

Processos Fonológicos	Sexo (%)			Grupos (%)			Queixa de Linguagem (%)		
	Masc	Fem	p-valor^a^	AIG	PIG	p-valor^a^	Sim	Não	p-valor^a^
Plosivação de fricativas	0,0	5,9	0,290	0,0	4,2	0,480	12,5	0,0	0,061
Posteriorização para palatal	10,5	11,7	0,907	0,0	16,7	0,139	37,5	3,6	0,008*
Frontalização de palatal	10,5	0,0	0,175	0,0	8,3	0,310	25,0	0,0	0,007*
Simplificação de líquida	26,3	5,8	0,105	8,3	20,8	0,350	50,0	7,1	0,005*
Simplificação de encontro consonantal	47,3	11,7	0,022*	25,0	33,3	0,614	75,0	17,9	0,002*
Simplificação da consoante final	31,6	17,6	0,342	8,3	33,3	0,107	75,0	10,7	0,000*
Ensurdecimento de plosivas	5,3	5,9	0,936	0,0	8,3	0,310	12,5	3,6	0,338
Ensurdecimento de fricativas	10,5	5,9	0,620	0,0	12,5	0,207	25,0	3,6	0,057
Africação	5,3	17,6	0,487	8,3	8,3	1,000	25	3,6	0,057
Processos de Substituição	42,1	11,8	0,045*	8,3	33,3	0,107	87,5	14,3	0,000*
Processos de Estruturação Silábica	23,5	17,6	0,063	0,0	33,3	0,025*	62,5	25,0	0,050

a Teste Exato de Fisher;

* p<0,005

Legenda: PIG = pequenos para idade gestacional; AIG = adequados para idade gestacional; Masc = masculino; Fem = feminino. Fonte: Própria

**Tabela 3:** Ocorrência dos processos fonológicos e a associação entre as variáveis do estudo na prova de imitação

Processos Fonológicos	Sexo (%)			Grupos (%)			Queixa de Linguagem (%)		
	Masc	Fem	p-valor^a^	AIG	PIG	p-valor^a^	Sim	Não	p-valor^a^
Posteriorização para palatal	10,5	5,9	0,620	0,0	12,5	0,207	37,5	0,0	0,001*
Frontalização de palatal	10,5	0,0	0,175	0,0	8,3	0,310	25,0	0,0	0,007*
Simplificação de líquida	31,6	5,9	0,055	8,3	25,0	0,240	62,5	7,1	0,001*
Simplificação de encontro consonantal	57,9	35,3	0,181	58,3	41,7	0,352	100,0	32,1	0,001*
Simplificação da consoante final	26,3	11,8	0,278	8,3	25,0	0,240	75,0	3,6	0,000*
Ensurdecimento de plosivas	5,3	5,9	0,936	0,0	8,3	0,310	12,5	3,6	0,338
Ensurdecimento de fricativas	10,5	11,8	0,907	8,3	12,5	0,712	25,0	7,1	0,162
Africação	10,5	0,0	0,175	0,0	8,3	0,310	25,0	0,0	0,007*
Processos de Substituição	47,4	29,4	0,277	16,7	29,2	0,421	100,0	21,4	0,000*
Processos de Estruturação Silábica	36,8	47,4	0,393	0,0	20,8	0,093	62,5	17,9	0,014*

a Teste Exato de Fisher;

* p<0,005

Legenda: PIG = pequenos para idade gestacional; AIG = adequados para idade gestacional; Masc = masculino; Fem = feminino. Fonte: Própria

**Tabela 4:** Análise das omissões e substituições nos encontros consonantais e consoantes finais entre os grupos e nas provas de nomeação e imitação

Tipos de ocorrência		Nomeação			Imitação		
		PIG (%)	AIG (%)	p-valor^a^	PIG (%)	AIG (%)	p-valor^a^
Omissão	CF	18,8	8,3	0,654	22,9	8,3	0,607
	GC	25,6	2,4		19,4	1,4	
Substituição	CF	2	4,1	0,327	10,4	8,3	0,608
	GC	11,3	11,4		9	18,8	

a Teste Exato de Fisher;

Legenda: PIG = pequenos para idade gestacional; AIG = adequados para idade gestacional; CF = consoante final; GC = grupo consonantal. Fonte: Própria

Consideraram-se significantes os resultados relacionados com a maioria dos PF e a queixa inicial de linguagem apresentada pelos pais na entrevista, nas tarefas de nomeação e imitação. Com relação às outras variáveis, verificou-se ausência de associação (Tabelas 2 e 3).

Quanto à análise das frequências de ocorrências dos processos fonológicos nas provas de imitação e nomeação entre os grupos, não foi encontrada diferença estatística, porém observou-se que a média de ocorrência para todos os processos presentes na população, apresentou sempre um maior percentual no grupo PIG.

Quanto à presença de PF produtivos, dos 14 processos analisados na prova de nomeação, o grupo PIG apresentou alteração em oito processos. Na presente pesquisa, adicionou-se o PF de africação, não observado no desenvolvimento típico infantil, totalizando nove processos alterados. O grupo AIG apresentou alteração em apenas três processos. Em ambas as provas, destaca-se a maior prevalência no grupo PIG, exceto no processo SEC, na prova de imitação (Figura 1).

**Figura 1:** Comparação e ocorrência dos processos fonológicos produtivos na prova de nomeação e imitação entre os grupos PIG e AIG

Legenda: PIG = pequenos para idade gestacional; AIG = adequados para idade gestacional; N = prova de nomeação; I = prova de imitação; RS = redução de sílaba; HC = harmonia consonantal; PF = plosivação de fricativa; PV = posteriorização para velar; PP = posteriorização para palatal; FP = frontalização de palatal; FV = frontalização de velar; SL = simplificação de líquida; SEC = simplificação do encontro consonantal; SCF = simplificação da consoante final; SP = sonorização de plosiva; SF = sonorização de fricativa; EP = ensurdecimento de plosiva; EF = ensurdecimento de fricativa; AFC = africação. Fonte: Própria

A Tabela 4 analisa o tipo de ocorrência mais frequente considerando as omissões e substituições dos fonemas líquidos laterais e não laterais, nas estruturas silábicas mais complexas constituídas de encontro consonantal e consoante final. Identificou-se o predomínio de omissões dos fonemas líquidos laterais e não laterais no grupo PIG nos encontros consonantais e nas consoantes finais.

## DISCUSSÃO

De acordo com os resultados encontrados, verifica-se que a população estudada apresenta significativamente maior número de processos fonológicos que alteram a estrutura da sílaba quando comparada ao grupo AIG, além de apresentar maior uso de PF produtivos e não esperados para a idade. Salienta-se que alterações no aspecto fonológico podem ser consideradas uma das mais evidentes no desenvolvimento infantil. Neste contexto, o diagnóstico e a intervenção precoce tornam-se imprescindíveis antes que se instalem intercorrências na aprendizagem da leitura e escrita e implicações psicossociais que interferem diretamente na percepção negativa de falante da língua materna.

Esses dados sugerem que diferenças com relação à presença de processos fonológicos que alteram a estrutura silábica entre os grupos PIG e AIG podem ser justificadas pela diferença de idade entre os grupos, como também por questões fisiopatológicas na população estudada, que parecem limitar a produção de palavras com maior exigência articulatória, visto que os erros do encontro consonantal no grupo PIG foram predominantemente por omissão, e não por substituição, como no grupo AIG (Tabela 4). Estudo recente^(14)^ ressaltou que crianças PIG que tiveram RCIU apresentaram alterações no desenvolvimento cognitivo quando comparadas com crianças AIG, o que poderia justificar as possíveis alterações no desenvolvimento fonológico, principalmente com relação à organização de estruturas silábicas mais complexas dentro da palavra, sendo um processo que envolve a interação de habilidades linguísticas, auditivas e cognitivas, e não exclusivamente a habilidade articulatória.

Uma possível disfunção placentária expõe o bebê à desnutrição, à diminuição da taxa de crescimento e provavelmente afeta os processos de desenvolvimento do cérebro, desencadeando alterações estruturais e funcionais, situações comumente observadas em bebês que tiveram RCIU associado ou não à prematuridade, o que potencialmente ressalta o surgimento de processos neurológicos e cognitivos alterados^(23)^, que podem interferir no processamento fonológico^(11)^. Outro estudo^(13)^ relatou existência de evidências limitadas de que as crianças com RCIU que nasceram PIG a termo podem ter problemas com atenção, impactando a aprendizagem e o comportamento à medida que avançam na escola, sugerindo que esta seria uma área útil para trabalhos futuros.

No presente estudo, as crianças foram classificadas como PIG ao relacionar peso e IG logo após o nascimento. Como essa classificação é amplamente discutida na literatura por conter indefinições fisiopatológicas, nesta pesquisa, como em outras relacionadas ao tema, encontra-se a limitação na classificação e na definição se a criança é PIG por ter sofrido uma RCIU ou se é constitucionalmente pequeno, por questões genéticas e/ou fisiológicas que determinam essa condição, e não por questões patológicas.

Os processos fonológicos de SEC e SCF foram os mais presentes na população estudada, corroborando estudos^(24-26)^ que também relataram que por envolver estruturas silábicas mais complexas são os últimos a desaparecerem. A idade máxima prevista para a eliminação desses processos, de acordo com o teste utilizado, seria sete anos^(17)^, sendo que estudos alertam para o desaparecimento mais precoce^(8,25,27)^. Ressalta-se, inclusive, o estudo realizado com crianças soteropolitanas^(28)^ e outros referem a possibilidade de influência da variação linguística na aquisição fonológica, interferindo na idade de eliminação desses processos^(7,21,29)^.

Analisando a faixa etária investigada, espera-se que ocorra a finalização do período aquisicional com a estabilização dos erros e predomínio da precisão fonológica a partir dos cinco anos^(8,24,28)^. No grupo PIG, constatou-se média de idade inferior ao grupo AIG, porém acima dos cinco anos nos dois grupos. Mesmo diante dessa diferença, o número de processos produtivos não esperados para a idade é mais acentuado no grupo PIG, como visualizado na Figura 1.

Identificou-se a presença de PF como plosivação de fricativa e simplificação de líquidas, que de acordo com o teste utilizado a idade de desaparecimento é entre dois a três anos e meio, além da posteriorização e frontalização de palatal, em que a eliminação ocorre por volta dos quatro anos e meio^(17)^. Destaca-se também a presença de PF que não são comuns no desenvolvimento fonológico típico, como o PF de ensurdecimento, e no caso específico dessa amostra ocorreu também à presença do PF de africação.

Estudo realizado em 2012^(26)^ encontrou que os processos fonológicos de SEC e SCF estiveram presente na faixa etária entre sete anos e sete anos e 11 meses, acentuadamente, nas crianças de classe econômica baixa. Os autores do presente estudo questionam a presença de PF em idades mais avançadas, já que a condição ideal, principalmente no primeiro ano do ensino fundamental I, que corresponde à faixa etária de seis e sete anos, é que as crianças não apresentem qualquer tipo de troca fonológica na sua fala, pois a escrita das palavras apoia-se inicialmente na oralidade^(30)^. Isso reforça a necessidade de detecção e intervenção precoce das alterações de fala, independente da população investigada.

Ressalta-se que os grupos não foram pareados com relação à classe social e sexo, todavia ambos os grupos apresentaram exposição similar aos aspectos socioeconômicos e culturais, considerados importantes fatores de risco que influenciam o desenvolvimento fonológico^(24)^. Somado a isso, não houve associação entre essas variáveis e os grupos.

Na amostra, observaram-se distribuições semelhantes entre os sexos, contudo maior proporção de alteração fonológica foi encontrada no sexo masculino, comprovando, assim, outros estudos^(3,7,24)^. Com relação ao processo fonológico SEC na prova de nomeação, notou-se que a ocorrência foi significativamente mais presente nos meninos do que nas meninas (p = 0,022), discordando do estudo^(31)^ que observou uma tendência dos meninos apresentarem maior precisão fonológica.

Detectou-se associação entre a queixa de possíveis alterações na fala das crianças e a maioria dos processos fonológicos (Tabela 2), confirmando que os pais percebem a presença dos PF. Esses pais parecem identificar a produção de fala como inadequada, apesar de aceitável, ou comum, no seu meio social por, na maioria das vezes, não interferir na compreensão da mensagem^(21,29)^, como acontece com os PF de SL, SEC e SCF, que apresentaram maior ocorrência na população estudada. Acredita-se que essa percepção dos pais está relacionada à busca do padrão de fala culturalmente aceito e que caso a criança alcance ela tenha possibilidades de mudanças que favorecerão o enfrentamento de outros espaços comunicativos, sem correr o risco da sociedade rotular os seus filhos como aqueles que “não sabem falar ou que falam errado”. Entende-se que alcançar um padrão de fala mais adequado ameniza o sofrimento psicossocial e emocional^(1)^, o que possivelmente esses pais buscam para os seus filhos.

Em relação ao achado do grupo AIG apresentar a média do PF de SEC maior do que do grupo PIG e os tipos de erros, percebeu-se que o predomínio é de substituição da líquida lateral /l/ pela líquida não lateral /ɾ/, levando a pensar que seria de fato uma variação linguística, e não uma falta de habilidade na produção de sílabas mais complexas, corroborando com outros autores^(21,27)^.

Esse tema ainda é pouco explorado, sendo necessários estudos com amostras mais significativas, abrangendo outros aspectos da linguagem, além do fonológico.

Os resultados deste estudo servem de alerta aos profissionais de saúde que atuam com essa população quanto à necessidade da prevenção, identificação e intervenção precoce dos transtornos da fala visando à comunicação saudável e à qualidade de vida. É fundamental também maior atenção à saúde da comunicação na primeira infância com implementação de políticas públicas que incluam as crianças que nascem a termo e são classificadas como PIG a fim de evitar prejuízos futuros nas áreas que afetam o desenvolvimento cognitivo, linguístico, psicológico e social.

Embora não se tenha observado associação entre alterações fonológicas e crianças PIG, ocorreram particularidades em alguns aspectos fonológicos dessa população, o que merece ser investigado mais profundamente, incluindo estudos com maior número de crianças, visto que uma limitação importante foi o reduzido número amostral dessa coorte. Isso impossibilitou, inclusive, o pareamento por idade. Acredita-se que com uma amostra maior as evidências relacionadas ao número de processos produtivos mais acentuado no grupo PIG sejam confirmadas estatisticamente.

Outra consideração é a classificação utilizada não possibilitar diferenciar as crianças que são PIG por apresentar algum tipo de sofrimento durante o período intrauterino daquelas que são pequenas por questões genéticas e/ou fisiológicas, excluindo qualquer condição patológica.

## CONCLUSÃO

No presente estudo, observou-se que crianças PIG apresentaram significativamente maior número de processos fonológicos que alteram a estrutura da sílaba quando comparadas àquelas do grupo AIG. Os processos fonológicos presentes e não esperados para idade na população PIG foram plosivação de fricativa, simplificação de líquidas, posteriorização e frontalização de palatal, ensurdecimento de plosivas e fricativas, além da simplificação do encontro consonantal e simplificação de consoante final, que foram os de maior ocorrência em ambos os grupos. Não foi encontrada associação entre alterações fonológicas e crianças PIG, contudo se identificou maior uso de processos fonológicos produtivos nesse grupo. Destaca-se a importância de um olhar diferenciado para essa população a fim de detectar precocemente alterações relacionadas aos aspectos da linguagem oral como a fonologia.
